# Quality Assurance and Quality Control in the Global Trachoma Mapping Project

**DOI:** 10.4269/ajtmh.18-0082

**Published:** 2018-07-23

**Authors:** Anthony W. Solomon, Rebecca Willis, Alexandre L. Pavluck, Wondu Alemayehu, Ana Bakhtiari, Sarah Bovill, Brian K. Chu, Paul Courtright, Michael Dejene, Philip Downs, Rebecca M. Flueckiger, Danny Haddad, P. J. Hooper, Khumbo Kalua, Biruck Kebede, Amir Bedri Kello, Colin K. Macleod, Siobhain McCullagh, Tom Millar, Caleb Mpyet, Jeremiah Ngondi, Benjamin Nwobi, Nicholas Olobio, Uwazoeke Onyebuchi, Lisa A. Rotondo, Boubacar Sarr, Oumer Shafi, Oliver Sokana, Sheila K. West, Allen Foster, for the Global Trachoma Mapping Project

**Affiliations:** 1 Clinical Research Department, London School of Hygiene & Tropical Medicine, London, United Kingdom;; 2 London Centre for Neglected Tropical Disease Research, London, United Kingdom;; 3 Task Force for Global Health, Decatur, Georgia;; 4 The Fred Hollows Foundation Ethiopia, Addis Ababa, Ethiopia;; 5 Berhan Public Health and Eye Care Consultancy, Addis Adaba, Ethiopia;; 6 Sightsavers, Haywards Heath, West Sussex, United Kingdom;; 7 ilimanjaro Centre for Community Ophthalmology, Division of Ophthalmology, University of Cape Town, Cape Town, South Africa;; 8 Michael Dejene Public Health Consultancy Services, Addis Ababa, Ethiopia;; 9 Emory Eye Center, Atlanta, Georgia;; 10 Orbis International, New York, New York;; 11 Blantyre Institute for Community Outreach, Blantyre, Malawi;; 12 Federal Ministry of Health, Addis Ababa, Ethiopia;; 13 Light for the World, Addis Ababa, Ethiopia;; 14 Sightsavers Nigeria, Kaduna, Nigeria;; 15 Department of Ophthalmology, Jos University, Jos, Nigeria;; 16 The Carter Center, Atlanta, Georgia;; 17 RTI International, Washington, District of Columbia;; 18 National Trachoma Control Program, Department of Public Health, Federal Ministry of Health, Abuja, Nigeria;; 19 Ministère de la Santé et de la Prévention Médicale, Dakar, Senegal;; 20 Eyecare Department, Ministry of Health, Honiara, Solomon Islands;; 21 Dana Center for Preventive Ophthalmology, The Wilmer Institute, Johns Hopkins School of Medicine, Baltimore, Maryland

## Abstract

In collaboration with the health ministries that we serve and other partners, we set out to complete the multiple-country Global Trachoma Mapping Project. To maximize the accuracy and reliability of its outputs, we needed in-built, practical mechanisms for quality assurance and quality control. This article describes how those mechanisms were created and deployed. Using expert opinion, computer simulation, working groups, field trials, progressively accumulated in-project experience, and external evaluations, we developed 1) criteria for where and where not to undertake population-based prevalence surveys for trachoma; 2) three iterations of a standardized training and certification system for field teams; 3) a customized Android phone–based data collection app; 4) comprehensive support systems; and 5) a secure end-to-end pipeline for data upload, storage, cleaning by objective data managers, analysis, health ministry review and approval, and online display. We are now supporting peer-reviewed publication. Our experience shows that it is possible to quality control and quality assure prevalence surveys in such a way as to maximize comparability of prevalence estimates between countries and permit high-speed, high-fidelity data processing and storage, while protecting the interests of health ministries.

## INTRODUCTION

Trachoma is the leading infectious cause of blindness.^1^ To help direct global elimination of trachoma as a public health problem by 2020,^2^ the Global Trachoma Mapping Project (GTMP) aimed to complete the baseline trachoma map worldwide.^3^ Technical, scientific, and financial oversight to the GTMP was provided through a complex network of partners with complementary mandates, skills, and capacities, including national governments, academic institutions, and nongovernmental organizations. A true international collaboration,^4^ the GTMP delivered high-quality^5^ population-based prevalence data on trachoma at unprecedented speed and scale.

Although the singular form of the word “project” is used in its title, the GTMP was actually a series of 55 trachoma mapping projects, each of which mapped between one^6–8^ and 91^9^ evaluation units (EUs) for trachoma. A project covered the trachoma mapping needs of a whole country, or of a regional state (Ethiopia) or state (Nigeria). In some projects, a phased approach was used, initially mapping a small number of EUs in which the likelihood of trachoma being a public health problem was felt to be the greatest, on the basis that mapping might be extended if prevalence was found to be high and not extended if it was not. Individual projects were owned and operated by health ministries or the local equivalent.^10,11^ Each EU was mapped using a population-based prevalence survey powered to be 95% confident of detecting an expected 10% prevalence of the sign “trachomatous inflammation—follicular”^12^ in 1- to 9-year olds, with absolute precision of 3% and a design effect of 2.65.^10^

The template methodology has been described in detail elsewhere.^10^ The present article documents the steps that were taken in each constituent project, and at global level, to adhere to the tenets of that template and to try to maximize the accuracy and application of the output. In the spirit of full disclosure, it also lists quality assurance and quality control measures that we did not take, either because doing so would have been too expensive or impractical or because the prompt to do so came with experience. Some measures in the latter category have been introduced for baseline, impact, and surveillance trachoma prevalence surveys supported by Tropical Data (www.tropicaldata.org),^13,14^ following the end of the GTMP.

## METHODS

Expert opinion, distilled through a series of teleconferences of the GTMP’s Methodologies and Prioritization Working Groups,^10^ was used to develop criteria for where to map and where not to map. We used computer simulation to confirm that population-based prevalence surveys were needed for mapping,^15^ rather than a quicker and epidemiologically dirtier approach. We held meetings and teleconferences of each of the four Working Groups (Methodologies, Prioritization, Tools, and Training), and convened the GTMP Advisory Committee to oversee development of pilot systems that were then trialed in the field in Oromia, Ethiopia, in October 2012.^10^ The training system, electronic data collection app and field methodologies were all subsequently refined and enhanced as a result of this experience.

The GTMP was formally launched on December 17, 2012, and supported trachoma prevalence survey fieldwork until January 19, 2016, operating in a total of 29 countries. It acquired and processed data on 625,541 households and 2,667,457 examined people. Improvements were progressively introduced during rollout, with identification of issues that warranted improvement facilitated by weekly teleconferences of the core GTMP team, 10 periodic meetings of the Advisory Committee, and formal midterm and end-of-project evaluations by (different) external consultants.

## RESULTS

The criteria used for determining where to map and where not to map are given in the Panel. The original field team training manual^16^ was superseded by two revisions: version 2 from May 24, 2013,^17^ and version 3 from August 15, 2014,^18^ with projects beginning after those dates using the updated versions. Quality assurance and quality control points used in the GTMP’s systems and methodologies are presented in a series of tables, covering issues relating to scope of mapping (Table 1); survey methodology (Supplemental Table 1), planning, budgeting, and logistics (Supplemental Table 2); training (Supplemental Table 3); survey implementation and field support (Supplemental Table 4); data entry (Supplemental Table 5); data management (Supplemental Table 6); data storage (Supplemental Table 7); and interpretation, reporting, and application of results (Supplemental Table 8). Common to all of these system details was a sequence of development through expertise and experience, consensus building, process design, operationalization, feedback, and follow-up. Measures were put in place through a combination of first-principles thinking (e.g., Supplemental Table 3, row 1), critical review of our own and others’ previous work (e.g., Supplemental Table 1, row 4), and progressively accumulated experience (e.g., Supplemental Table 2, row 2).

**Table 1:** Preemptive and corrective measures put in place by the GTMP to avoid pitfalls inherent in trachoma mapping: issues relating to the scope of mapping

No.	The GTMP…	…To reduce the impact of, or avoid…	…Which otherwise might have led to…	Examples of instances where this measure helped (or might have helped)
1	Systematically discussed countries (and administrative divisions within countries) with individuals who had local knowledge, in an effort to uncover available evidence for possible trachoma endemicity, with documentation of evidence, and action where needed	Lack of expressed need to map in areas where mapping is needed	Delay in identification of endemic areas, delay in elimination program initiation, and failure to achieve GET2020	The GTMP systematically discussed the need for trachoma surveys in the Democratic Republic of the Congo with key informants^19^ and the Ministère de la Santé^20^
		Lack of expressed need to map in areas where trachoma was historically found but has now disappeared	Continuing uncertainty and repeated reexamination of the same evidence over the need or otherwise to conduct mapping	In 1982, a study of prevalence and causes of blindness and low vision was conducted in eight provinces of Indonesia; trachoma was one of the top 10 causes; by 2013, trachoma had disappeared (unpublished Indonesia Ministry of Health data)
2	(Where evidence to justify mapping was of low quality) undertook mapping using a phased approach	Failure to take into account prevalence estimates in adjacent areas, as they accrued, in decision-making on whether there was a need to map	Excessive use of resources to document the absence of trachoma at baseline, or delay in identification of endemic areas, delay in elimination program initiation, and failure to achieve GET2020	The GTMP phased survey rollout in the Democratic Republic of the Congo,^20^ Yemen (manuscript in preparation) and Zimbabwe^21^
3	(Where evidence to justify mapping was completely absent but suspicion of trachoma existed) provided technical and financial support to undertake preliminary survey work to determine whether baseline population-based prevalence surveys were needed	Expressed need to map in areas where mapping was not needed	Excessive use of resources to document the absence of trachoma at baseline	The GTMP undertook preliminary survey work in Tanzania to rule out areas unlikely to have trachoma as a public health problem^22^
		Lack of expressed need to map in areas where mapping is needed	Delay in identification of endemic areas, delay in elimination program initiation, and failure to achieve GET2020	The GTMP undertook preliminary survey work in Papua New Guinea to provide evidence to justify population-based prevalence surveys^23^
4	Used a positive trachoma rapid assessment^24^ or other positive preliminary data on the presence of trachoma to initiate support for a population-based prevalence survey in the corresponding administrative area, as soon as possible	Assumption that data from a trachoma rapid assessment provide prevalence estimates	Maximally biased estimate of prevalence potentially used for programmatic decision-making	The GTMP did this for the duration of its operation
5	Initiated contact with health ministries of countries that may have been trachoma endemic (and responded to countries that reached out to us on learning about the GTMP), then engaged in discussions to determine whether mapping was needed	Countries being isolated from the international trachoma community	Delay in identification of endemic areas, delay in elimination program initiation, and failure to achieve GET2020	Colombia identified trachoma in communities in the Amazon rainforest, near to the border with Brazil, between 2003 and 2006,^25^ but limited international engagement occurred until the GTMP visited in 2013^26^
6	Undertook detailed discussions with health ministries over the benefits and risks associated with using the standardized systems and approaches of the GTMP for trachoma mapping, as opposed to completing trachoma mapping via other means	Incomplete uptake of standardized systems and approaches developed by the GTMP, and/or the incomplete use of funds allocated to the GTMP	Heterogeneity of approaches and/or failure to meet donors’ expectations	The GTMP did this for the duration of its operation
7	Channeled financial resources donated by bilateral organizations to undertake baseline trachoma mapping in any country where baseline mapping was justified	Domestic funds available to map insufficient to meet clear needs	Delay in identification of endemic areas, delay in elimination program initiation, and failure to achieve GET2020	A national survey of blindness, low vision, and trachoma in Ethiopia in 2005–2006^27^ showed that trachoma was highly and widely endemic in Oromia, the largest regional state. But by 2012, survey work had been undertaken in only 10 of Oromia’s then-current 265 rural districts.^28^ The GTMP supported mapping of the rest of the regional state^29^
8	Encouraged health ministries to piggyback collection of data on other diseases of local importance, advocated to funders to secure permission to do so, and provided technical support to adjust fieldwork protocols and data collection tools as needed	Co-endemic diseases with data needs not mapped with baseline trachoma surveys	Lost opportunity for achieving efficiencies in the use of human and financial resources	In two EUs of the Solomon Islands and one EU of Vanuatu, the GTMP collected population-based data on the prevalence of yaws and trachoma at the same time^6,30,31^
9	Supplemented hour-by-hour communication with weekly formal teleconferences of the core project group, to review progress and plan activities, country by country	Centralization of information and decision-making in the hands of one individual or one partner organization	Lost opportunities to benefit from complimentary experiences and to hear dissenting voices	The GTMP held weekly formal teleconferences for the duration of its operation

GET20202 = global elimination of trachoma as a public health problem by 2020; GTMP = Global Trachoma Mapping Project; EU = evaluation unit.

PANEL

Criteria for where to map and where not to map used by the GTMP, 2012–2016.

Where to map:

- where, on the basis of historical data on trachoma in that district, current data on trachoma in adjacent districts, socioeconomic conditions, and access to water and sanitation, the population is very likely to be trachoma endemic; or
- where trichiasis surgery is being performed by local health-care providers; or
- where individuals with trichiasis are presenting to local health-care providers; or
- where individuals with trichiasis are being identified as part of community outreach campaigns

Where not to map:

- where there is no justification to believe trachoma might be endemic, based on the previously discussed data; or
- where epidemiologically valid prevalence data collected within the last 10 years are already available; or
- where undertaking mapping might put field teams at a security risk; or
- where the responsible authorities, following in-depth discussions, do not prioritize elimination of trachoma as a public health problem

## 

## DISCUSSION

“An expert,” Niels Bohr is reported to have said, “is a person who has found out by their own painful experience all the mistakes that one can make in a very narrow field.”^32^ In that sense, we regard ourselves as approaching expert status in the conduct of population-based prevalence surveys in developing countries. The “painful experience” part of our journeys to this point means that this article was not written to give its authors an opportunity to claim epidemiological superiority over those who have designed, supervised, participated in, or paid for population-based trachoma surveys conducted outside the GTMP. During the course of our careers, we have scattered survey design flaws over the trachoma-endemic globe; we have tried to document those mistakes here. Within the GTMP, we still did not achieve perfection, having had to balance our desire to achieve it with the knowledge that doing so would have reduced efficiency. As a particular example, we are aware that GTMP field teams often failed to enumerate residents who were eligible to be examined but did not participate,^33^ despite the fact that our system facilitated it and our field team training system specified doing so. We think that the team members were anxious that they would face supervisors’ criticism if they achieved much less than 100% enrolment, which is a training and communication issue that we tried (and continue to try) to address; we believed that pausing fieldwork to alter enumeration habits would not have been productive. It should also be noted that a proportion of the potential problems that we list in the table as things that we attempted to avoid or correct in the GTMP are actually ghosts-of-problems-future that we have not necessarily yet encountered in real life. However, recognizing and confronting both previous failures and near-misses is important, and this article is an attempt to comprehensively catalog both the errors that we prevented or detected using the systems and methodologies of the GTMP and those that we continue to make and will try to eliminate, where possible, in the next phase^13,14^ of population-based data collection to guide trachoma elimination. If our experience can be used to help others strengthen the design and execution of their community-based surveys at the same time, it will be a double win.

Field-based surveys are complex undertakings, with many moving parts. We set out to generate a whole-of-process system with as few visible joins as possible, supporting survey implementation from the point of determining whether a survey was justified, through to interpreting health ministry-approved data and applying those data for the purposes of improving public health. In such a system, an error in the design or execution of one part of the process can have catastrophic effects on the project as a whole. Before the launch of the GTMP, therefore, we attempted to ensure that all phases of the implementation process had been planned to the fullest possible extent, with decisions made for one phase complementary to decisions made for the others; this article in part demonstrates the fruit of those efforts. As a high-profile endeavor within the neglected tropical diseases sector, within which there are many competing priorities, failure of the GTMP’s systems to work as promised might have had reputational consequences for progress against trachoma internationally.

In that context, implementation of a purely electronic data pathway from collection through to display and application carried some risk, both in terms of risk of failure of a system built specifically to serve the GTMP, and in terms of the challenge of convincing scores of health ministries and other partner organizations to simultaneously jump with us from paper to silicon. An occasional objection raised was that without paper forms, we would not have the “original record” and would, therefore, be unable to investigate apparent problems in the data; this objection ignores the fact that irremediable errors are also made when recording data on paper, including many (such as skipped fields, out-of-range values, and illegible handwriting) that our app prevented by design. We believe that our recorders’ error rate (estimated on the basis of the data on trichiasis in children—all reports of which were verified [Supplemental Table 4, row 3]—at 1.4 errors per 10,000 keystrokes) compares favorably with previously published data on error rates of data entry operators. Rabbitt found that when individuals were asked to electronically record answers to a question with two possible responses (an analogy from our surveys would be, “Is there trichiasis in the right eye?”), the observed error rate was 60 per 10,000.^34^ An outstanding question is whether estimates of trachomatous trichiasis prevalence in adults should be automatically corrected downward to account for the inevitability of these occasional errors, on the basis that when recording the presence or absence of a rare event, erroneous entries are considerably more likely to bias prevalence estimates upward than downward.

The aforementioned question may leave the impression that we felt that we engaged in a high-stakes game by setting up to complete the GTMP and choosing electronic data capture. We would, therefore, be remiss if we failed to acknowledge that (other than in terms of scale and standardization) the GTMP was the setting for an evolution rather than a revolution in trachoma surveys. Our collective efforts outlined here owe much to others.^24,35–40^ We think we have built on that previous work by making electronic data capture the emerging standard for neglected tropical disease epidemiology, by highlighting the need for certification of clinical examination accuracy in field surveys, by emphasizing data quality, and by the measures that we have undertaken to ensure local ownership.^5,11^

Supporting health ministries to fulfill their mandate to lead and encouraging appropriate contributions and buy-in from all relevant stakeholders are extremely important issues in their own right.^11^ They are also an important step to quality assure future prevalence surveys (which will be required to assess the impact of interventions on progress toward elimination^41^) because increasing local capacity creates more equal partnerships that will be primed to work together on robust survey designs in the next round.

We are open to constructive criticism from and future collaboration with others and look forward to continuing to adapt and improve as we work toward a world in which surveys to estimate the prevalence of trachoma eventually become unnecessary.^42^

## Supplementary Material

Supplemental tables
