# Supplementary material for: Quality Assurance and Quality Control in the Global Trachoma Mapping Project
Source: Am J Trop Med Hyg. 2018 Jul 23;99(4):858–63. doi: 10.4269/ajtmh.18-0082 (PMC6159583; doi:10.4269/ajtmh.18-0082)
Supplement: Supplementary file 1 [file tpmd180082.SD1.pdf]

Quality Assurance and Quality Control in the Global Trachoma Mapping Project

SUPPLEMENTAL TABLE 1

Pre-emptive and corrective measures put in place by the GTMP to avoid pitfalls inherent in trachoma mapping: issues related to survey methodology

	**The GTMP…**	**…to reduce the impact of, or avoid…**	**…which otherwise might have led to…**	**Examples of instances where this measure helped (or might have helped)**
1	asked countries to prepare a first draft of the survey protocol	local adoption of a methodology without local understanding of why each of its elements was important	failure to build local capacity.	The GTMP did this in each of its constituent projects.
		imposition of locally inappropriate survey elements if local partners feel inhibited about challenging a template	difficulties in survey implementation.	
2	ensured that the draft survey protocol was consistent with WHO recommendations, working with the health ministry and local partners to refine the draft as needed	international inconsistency	prevalence estimates that could not be compared between settings.	The GTMP did this in each of its constituent projects.
3	assisted countries to frame EUs of appropriate sizes (generally at the level of the local administrative unit for health care management and ideally containing	framing of inappropriately large EUs	potential to miss significant pockets of disease.	In Yobe State, Nigeria, a previous population-based trachoma prevalence survey covered a population of > 2 million people in

	populations of 100,000–250,000 persons[35])			a single EU.[43]
		framing of inappropriately small EUs	excessive use of resources for mapping, or extrapolation of results from a small EU to provide prevalence estimates for a larger population.[44]	In the Solomon Islands, the regions of Rennell-Bellona (estimated population 3041) and Temotu (estimated population 21 362) had sufficiently similar socio-economic and environmental characteristics to be combined to form a single EU.[31]
4	assisted countries to design epidemiologically valid cluster selection methods within EUs	selection of too many clusters	excessive use of resources for mapping.	Due to a misunderstanding, twice the required number of clusters were selected and visited in one GTMP-Mozambique EU that had been formed by combining 2 adjacent districts. We subsequently included explicit discussion of the implications of EU formation on cluster selection in our conversations with health ministries.
		selection of too few clusters	potential to miss significant pockets of disease.	In some trachoma prevalence surveys conducted prior to the GTMP, 7–14 clusters were selected for inclusion.[45–47]
		selection of clusters using a biased	generation of inaccurate	All GTMP-supported surveys applied epidemiologically-

		methodology	prevalence estimates.	appropriate cluster selection methods.
5	assisted countries to design epidemiologically valid household selection methods within clusters	selection of too many households	excessive use of resources for mapping, either through enrolment of an excessive total number of households per EU, or through making the workload required in one cluster too great to be reliably completed by one team in a single day.	In 1999, in order to select an appropriate sub-village for a longitudinal study, AWS and his team examined 5527 of 5703 residents (some or all residents of 1099 of 1103 households) of a single trachoma-endemic village in Tanzania,[48] for reasons that he is now unable to fully explain. It took him and one other experienced grader nearly 3 months of full-time work.
		selection of too few households	failure to achieve an appropriate sample size, or inefficiency through having field teams unproductive for long periods of each day.	All GTMP-supported surveys involved selection of 25–45 households per selected cluster.
		selection of households using a biased methodology	generation of inaccurate prevalence estimates.	For the first few clusters enrolled in the Democratic Republic of the Congo, teams only visited households that had children; this was noted by our data manager and corrected via a telephone call.
6	selected a fixed number of households per cluster, rather than a fixed number of	perceived pressure to enrol individuals causing coercion of	abrogation of ethical	The GTMP did this in each of its constituent projects, except in Viet

	individuals	cluster residents or biased sampling, particularly towards the end of the day in the field	responsibilities, or selection bias.	Nam.[49]
7	developed a standard smartphone app for data collection and supplied it for use in all countries, allowing any changes in practice to be quickly incorporated through the app	lag in practice behind policy, which lags behind learning	continued implementation of known flaws.	The GTMP's purpose was to complete baseline trachoma mapping, but it soon received requests to support impact and surveillance surveys[41] for trachoma, too. At these phases of programme evolution, the prevalence of trichiasis "unknown to the health system[a]"[35] is important. This led rapidly to the incorporation into the standard survey of questions about previous management in eyes recorded as having trichiasis.[18]
8	designed surveys to include collection of global positioning system data from each household enrolled	failure to include independently verifiable geolocation of selected households and clusters	inability to ensure that households and clusters have been appropriately enrolled.	In one constituent project of the GTMP, one team logged all households in 2 clusters at a single location in the national capital, several hundred kilometres from the EU ostensibly being surveyed. Following further investigation on the ground by the health ministry, those data were rejected and the

[a] This excludes cases that have already been operated on, for which operations have been refused, or which are already scheduled to receive operations.

				team members redeployed away from the GTMP.
9	enrolled, as standard, all residents aged ≥1 year in each household selected for inclusion	failure to examine adults (or examination of adults only if they lived in the same house as 1–9-year-olds[50])	inability to estimate a meaningful prevalence (or generation of a potentially biased estimate of the prevalence[50]) of trachomatous trichiasis.	Within the GTMP, 3 EUs in Chad and 3 EUs in Egypt had to be re-surveyed because field teams only examined 1–9-year-olds; in Cambodia, only households in which 1–9-year-olds lived were enrolled[50]; in Viet Nam, only 1–9-year-olds and ≥50-year-olds were enrolled.[49]
		failure to examine children[47]	inability to estimate a meaningful prevalence of trachomatous inflammation—follicular.	The GTMP did this in each of its constituent projects.
		creation of an incentive for household residents very keen to be examined, or very keen not to be examined, to misrepresent their age	bias in prevalence estimates.	In recent trials of a trachomatous trichiasis-only survey methodology, when only those aged ≥40 years were examined, unexpectedly large numbers of individuals claiming to be aged 40–45 years were enrolled.[51]
10	supported health ministries to obtain local ethical clearance before surveys started	neglect of locally important ethical considerations in survey design	failure to “take into consideration the laws and regulations of the country or countries in which the research is to be performed as well as applicable international norms	The GTMP did this in each of its constituent projects.

			and standards", as required by the Declaration of Helsinki.[52]	

SUPPLEMENTAL TABLE 2

Pre-emptive and corrective measures put in place by the GTMP to avoid pitfalls inherent in trachoma mapping: issues related to planning, budgeting and logistics

	**The GTMP…**	**…to reduce the impact of, or avoid…**	**…which otherwise might have led to…**	**Examples of instances where this measure helped (or might have helped)**
1	closely reviewed budgets against the agreed methodology and a standard budget template and ensured that the methodology was consistently reflected in the budgeting assumptions[53]	essential activities omitted, or excessive resources requested to undertake mapping	mapping activities not aligned with agreed methodology, inefficient use of resources, or shortfall in funding with consequent failure of one or more of the GTMP's constituent project.	Resources to support field team supervisors added to budgets in several projects. In one country, excessive requests were trimmed, resulting in a budget reduction of 28%.
2	provided Android smartphones for survey teams, with (if possible) survey forms already pre-loaded	possible local purchase of phones with outdated versions of the Android operating system and/or difficulties in ensuring correct software installation	delays in commencing surveys, or reversion to the use of paper-based data collection.	The GTMP tried to do this in each of its constituent projects. Where it did not (because, for example, it was difficult to import phones, or lead times were too short), phone cost was often higher, and internet bandwidth occasionally made it challenging to download survey software.

3	provided binocular, 2.5× magnifying loupes for graders to use	lack of provision of loupes by programmes; or provision of loupes that were uncomfortable to wear, prone to breakage, or of the wrong magnification	failure to use loupes, or use of loupes with the wrong magnification, leading to reduction in diagnostic accuracy.	The GTMP did this in each of its constituent projects.

SUPPLEMENTAL TABLE 3

Pre-emptive and corrective measures put in place by the GTMP to avoid pitfalls inherent in trachoma mapping: issues related to training

	**The GTMP…**	**…to reduce the impact of, or avoid…**	**…which otherwise might have led to…**	**Examples of instances where this measure helped (or might have helped)**
1	provided a standard training system for graders and recorders,[18] and required individuals to be certified in its use before using it to train others[10]	different training systems, or different interpretation of the same training system, delivered to teams in different projects; and duplication of effort	variable quality training.	Over the course of its 3-year operation, the GTMP used 3 successive versions of a standardized training system.[10]
2	provided all elements of the standard training system in English, French, Arabic, Spanish and Portuguese,[18] having had the accuracy of translation in each case checked by ophthalmologists with that language as a mother tongue	differential attention to quality control and quality assurance in different settings, depending on language preference	variable quality training.	All 3 iterations of the GTMP's standardized training system were made available in 5 languages.[10]
3	trained local trachoma grader trainers, or (where there were insufficient human resources to meet the local training need) identified and funded GTMP-certified grader trainers from elsewhere to participate in training	human resources available to train local staff insufficient to meet the agreed need	delay in identification of endemic areas, delay in elimination programme initiation, and failure to achieve GET2020.	The GTMP funded a GTMP-certified grader trainer from the Solomon Islands to visit Vanuatu to train and certify local personnel as trachoma graders[6]; there were many other instances of similar collaboration within the GTMP.

4	assisted newly GTMP-certified trainers undertaking their first GTMP training week by providing a fellow trainer with previous experience of delivering GTMP training	trepidation or uncertainty	Inconsistency in field team training between sites.	The GTMP tried to do this in each of its constituent projects; it was not possible in some instances because of travel restrictions.
5	(where there was an absolute shortage of medical and paramedical personnel to train as graders) brokered secondments of GTMP-certified graders from elsewhere to assist with mapping	understaffed field teams	scarce personnel deployed to the field for long periods to complete surveys, or mapping not attempted at all.	The GTMP did this on several occasions; the details are somewhat politically sensitive.
6	insisted on strict adherence to the definitions of signs in the WHO simplified trachoma grading scheme[12]	often: over-diagnosis, because graders do not want to ignore obvious pathology; this stems from confusion between the role of a grader contributing to a prevalence survey (where definitions must be clear cut), and the role of a clinician caring for an individual (where knowledge of the natural history of disease and factors other than the patient's clinical signs contribute to formulating the management plan)	overestimation of the prevalence of trachoma, potentially leading to inappropriate allocation of resources for trachoma elimination.	In Togo in 2009, graders classified individuals with fewer than 5 follicles as having TF.[54]
7	undertook all grader training in known trachoma-endemic areas, and incorporated examination in the field as	deployment of graders who may not have previously had an opportunity to examine real subjects with the signs they are	uncertainty about grader competence.	The GTMP supported grader trainees from Lao People's Democratic Republic[59] and Cambodia[50] to be trained in

	part of the training process	then asked to identify[55–58]		Ethiopia, at the invitation of the Oromia Regional Health Bureau.
8	only deployed graders who had demonstrated their trachoma grading competency through formal inter-grader agreement exercises on real subjects, using the assessments of a GTMP-certified grader trainer[10] as the gold standard	deployment of graders whose competency had been assessed only through grading of slides or photographs of trachoma[55–58,60]	uncertainty about grader competence.	The GTMP did this in each constituent project, other than Viet Nam,[49] where travel of grader trainees to a more highly endemic country could not be undertaken. Though not allowing previously-experienced graders who did not pass the test to continue was controversial at the beginning, it was subsequently seen as an important demonstration of how important quality was to the GTMP. In some contexts, however, managing disappointed grader trainees became an issue in its own right.
9	set the standard for passing the inter-grader agreement exercise as a kappa of ≥0.70 for the presence or absence of the sign "trachomatous inflammation—follicular" in children aged 1–9 years	use of percentage agreement with the grader trainer,[43,46,61–63,64] or an unspecified measurement,[45,65] as the criterion for diagnostic accuracy	uncertainty about grader competence.	The GTMP did this in each of its constituent projects, other than Viet Nam (see above).
10	made the training system available for countries engaged in impact surveys for trachoma, even if they were conducted	different training systems delivered to teams in different settings; and duplication of effort to create	variable quality of training.	The GTMP training system was made available to India, Mali and Nepal for use in trachoma prevalence surveys that health

	without other involvement of the GTMP	training systems		ministries in those countries had planned to implement independently.

SUPPLEMENTAL TABLE 4

Pre-emptive and corrective measures put in place by the GTMP to avoid pitfalls inherent in trachoma mapping: issues related to survey implementation and field support

	**The GTMP…**	**…to reduce the impact of, or avoid…**	**…which otherwise might have led to…**	**Examples of instances where this measure helped (or might have helped)**
1	deployed in-service supervisors, each of whom were required to first pass the formal inter-grader agreement exercises on real subjects, using as the gold standard the assessments of a GTMP-certified grader trainer	lack of appropriate supervision[b]	drift in accuracy of grading over time; errors in application of fieldwork protocol; or unreported social, economic, health or supply issues that could adversely affect field team performance.	The GTMP did this in each of its constituent projects.
2	rapidly reviewed raw data	over-estimation of the mean number of residents per household in available census data	failure to examine a number of individuals in each EU that would permit calculation of prevalence estimates with acceptable precision.[c]	In Southern Nations, Nationalities and Peoples' Region of Ethiopia,[66] we requested that 4 clusters be added to an EU because there were too few 1–9-year-olds examined.

[b] We intend to further improve the standard and consistency of supervision in trachoma impact and surveillance surveys, through the use of a dedicated training package for supervisors

[c] We did not keep a sufficiently close eye on this issue early in project implementation. (In Oromia, for example, there were 2 EUs in which 651 and 653 1–9-year-olds were initially examined, but because of delays in data upload, teams had moved to other zones before this came to light.) Pre-GTMP surveys which stipulated a given number of subjects to be examined per cluster did not run this risk, but instead risked biased selection.

		under-estimation of the mean number of residents per household in available census data	examination of more individuals in each EU than necessary to permit calculation of prevalence estimates with acceptable precision, leading to inefficient use of resources.[d]	In Guinea, where 23 clusters were included per EU, the range of 1–9-year-olds examined per EU was 1113–3137.
3	telephoned field supervisors as soon as a record of trachomatous trichiasis in a child was identified	erroneous recording of the presence of trachomatous trichiasis in a child	potential mobilisation of a paediatric ophthalmologist or oculoplastic surgeon to provide service; or if undetected, tacit encouragement of a lack of concentration in the field.	In raw data from 55 projects, 519 cases of trichiasis were reported amongst 1 146 644 1–9-year-olds; 249 of those cases were confirmed when checked with field teams.
4	discussed and resolved fieldwork problems as they arose	uncertainty, confusion, inconsistency between teams	reductions in the accuracy and/or repeatability of prevalence estimates.	The GTMP did this in each of its constituent projects.

[d] We did not do this well enough. Pre-GTMP surveys which stipulated a given number of subjects to be examined per cluster did not run this risk, but instead risked biased selection.

SUPPLEMENTAL TABLE 5

Pre-emptive and corrective measures put in place by the GTMP to avoid pitfalls inherent in trachoma mapping: issues related to data entry

	**The GTMP…**	**…to reduce the impact of, or avoid…**	**…which otherwise might have led to…**	**Examples of instances where this measure helped (or might have helped)**
1	undertook all primary data recording in electronic format, using a purpose-built Android smartphone app, LINKS-GTMP[10,36]	failure to ensure high-fidelity transfer of data from paper to electronic format for the purposes of data analysis, and/or long delays while paper-based surveys are managed (photocopied, double entered, etc.)	data used to generate prevalence estimates not reflecting the findings observed in the field, and/or long intervals between survey completion and programmatic decision-making.	The GTMP did this in each of its constituent projects.
2	ensured that the LINKS-GTMP app did not permit fields to be skipped[e]	failure to collect or record available data	missing data, and uncertainty in analyses.	The GTMP did this in each of its constituent projects.
3	included "don't know" and/or "other" options in all multiple choice questions	data recorders being forced to stop, enter junk data or use a parallel reporting system	junk data, loss of system integrity or inability to proceed.	The GTMP did this in each of its constituent projects.
4	used check screens requesting recorders to verify data just entered	lack of flagging of entry of rare outcomes, such as trachomatous	missed opportunities to correct erroneous keystrokes at source.	The GTMP did this in each of its constituent projects.

[e] The dangers inherent in allowing skip fields are illustrated by the GPS data collected (for each household) in the GTMP. Because GPS signals are sometimes difficult or impossible to access, LINKS-GTMP allows recorders to proceed to the next question without collecting GPS coordinates, which teams were instructed to press only after the Android had tried to triangulate its location for at least 60s without success. In one project, a group of recorders rapidly developed a habit of always skipping the GPS field. In our next-generation app developed for Tropical Data, skipping GPS data collection is only possible after system-driven timeout.

		trichiasis		
5	restricted responses to sensible ranges – age, for example, could only be recorded as 1–100 years; for those reporting an age at last birthday of > 100 years, 100 years was recorded	errors	potentially uninterpretable data.	The GTMP did this in each of its constituent projects.

SUPPLEMENTAL TABLE 6

Pre-emptive and corrective measures put in place by the GTMP to avoid pitfalls inherent in trachoma mapping: issues related to data management

	**The GTMP…**	**…to reduce the impact of, or avoid…**	**…which otherwise might have led to…**	**Examples of instances where this measure helped (or might have helped)**
1	undertook data cleaning centrally (by BKC from 12/2012–06/2013; by RW from 07/2013–07/2015; by RW and AB from 07/2015–01/2016; identical algorithms were used throughout)	inconsistency and/or lack of objectivity in data cleaning	random error or bias.	The GTMP did this in each of its constituent projects.
2	checked that clusters lay within the boundaries of the EU by comparing the mean GPS coordinates of the households	inclusion of data from clusters that were inadvertently selected from outside the boundaries of the EU	potentially inaccurate estimation of trachoma prevalence for the EU.	The GTMP did this in each of its constituent projects.

	enrolled in a cluster with the EU shape-file[f]			
3	adjusted trachomatous inflammation—follicular prevalence data by age of subjects examined[10]	implied failure to recognise that those examined in a house-to-house survey may not necessarily be representative of the underlying population, because of absence, refusal, or illness	bias in estimates of prevalence.	The GTMP did this in each of its constituent projects.
4	adjusted trachomatous trichiasis data by age and gender of subjects examined [10]	implied failure to recognise that those examined in a house-to-house survey may not necessarily be representative of the underlying population, because of absence, refusal, or illness	bias in estimates of prevalence of trachomatous trichiasis, usually leading to overestimation.	The GTMP did this in each of its constituent projects.
5	equally weighted cluster-level outcome proportions when considering overall prevalence	over-emphasis of results on clusters with larger numbers or examined individuals	sampling or participation bias.	The GTMP did this in each of its constituent projects.
6	in projects commencing after 15 August 2014, classified trichiasis as trachomatous trichiasis if and only if the eyelid demonstrated easily visible conjunctival scarring [12], or could not be everted	implied failure to recognise that not all trichiasis is due to trachoma	misclassification bias in estimates of prevalence of trachomatous trichiasis, leading to overestimation.	Since adding collection of data on conjunctival scarring, 69% of all trichiasis recorded in GTMP surveys has been trachomatous trichiasis.
7	developed standard data analysis	human error	erroneous output.	The GTMP did this in each of its

[f] Accurate shape-files were often unavailable. In such cases, discussion with local staff was used to resolve uncertainties.

	algorithms to be run in R			constituent projects.
8	double-checked all output by eye	human error, or unintended consequences of the use of standard algorithms	erroneous outputs.	The GTMP did this in each of its constituent projects.
9	made the R code used freely available to any interested party (please see: https://github.com/itidat/tropical-data-analysis-public")	higher barrier to replication of analyses	scepticism.	For the R code, please see Supplementary information.
10	used a 2-stage health ministry data approval process and insisted on health ministry ownership of data [10]	lack of acknowledgement of national ownership of data	delay or failure in application of outputs to disease control.	The GTMP did this in each of its constituent projects. (The lack of an analogous process for routinely engaging, at the planning stage, government officials responsible for water, sanitation and hygiene (WASH) occasionally led to tensions over household-level WASH data, which were collected in nearly all constituent projects of the GTMP.[59, 60])

SUPPLEMENTAL TABLE 7

Pre-emptive and corrective measures put in place by the GTMP to avoid pitfalls inherent in trachoma mapping: issues related to data storage

	**The GTMP…**	**…to reduce the impact of, or avoid…**	**…which otherwise might have led to…**	**Examples of instances where this measure helped (or might have helped)**
1	programmed LINKS-GTMP to automatically upload data from micro-SD cards to a Cloud-located database, as soon as a data-enabled mobile phone network or WiFi network was detected	electronic data maintained in only one place, or in an insufficient number of places	need for repeat survey in the event of loss, damage or corruption of storage media.	The GTMP did this in each of its constituent projects.
2	applied 128-bit encryption at the transport layer,[10] and carried out subsequent data review, cleaning and approval only through a secure website with transport layer security, IP-restricted firewall, and site authentication and authorization, to which access could be gained only through password-protected login.	paper-based or electronic data containing personally identifiable information stored without adequate security	potential inadvertent disclosure of personally identifiable information on survey subjects, violating standard ethical principles for investigators.	The GTMP did this in each of its constituent projects; for Tropical Data,[13, 14] 256-bit encryption is being used, including encryption of data at rest.
3	went to extreme lengths to recover data stored on micro-SD cards that were, in the rare case, damaged in the field	loss of data and potential loss of faith in the reliability of data storage on Android smartphones	possible need to repeat surveys, and possible need to design a new system.	The GTMP lost data from 1 cluster (due to a lost Android), of a total of 12,631 clusters visited. Where Androids were damaged or corrupted, all stored data were

				recovered.

SUPPLEMENTAL TABLE 8

Pre-emptive and corrective measures put in place by the GTMP to avoid pitfalls inherent in trachoma mapping: issues related to interpretation, reporting and application of results

	**The GTMP…**	**…to reduce the impact of, or avoid…**	**…which otherwise might have led to…**	**Examples of instances where this measure helped (or might have helped)**
1	built capacity amongst collaborating partners in an effort to ensure that disaggregated data, if examined, were interpreted correctly	assumption that cluster-level data can be used to provide prevalence estimates for the cluster,[64] or for a subdivision of the EU[43]	decisions made using data not powered to provide the estimates generated.	The GTMP did this in each of its constituent projects.
2	calculated 95% confidence intervals for prevalence estimates by bootstrapping adjusted cluster-level proportions, with replacement, over 10,000 replications	confidence intervals around prevalence estimates not calculated, not calculated using an appropriate methodology, or not reported	assessment of precision of prevalence estimates made difficult.	The GTMP did this in each of its constituent projects.
3	provided epidemiologist support to local authors, as needed, to draft project-specific manuscripts once each project was complete; and brokered agreement to publish the resulting papers, if accepted under the journal's normal criteria, in a series of supplements to a peer-reviewed journal, with financial support from the project to make those	data not published in peer-reviewed journals	lower visibility of data and of local efforts.	There has been considerable interest in the published output of the GTMP, with a number of special issues of *Ophthalmic Epidemiology*[5,81] produced.

	papers open-access[8,9,22,23,29,31,50,59,66,69–80]			
4	automatically channelled prevalence category data to the open access website of the Global Atlas of Trachoma,[82,83] with explicit health ministry agreement, as soon as they were fully approved by the health ministry	prevalence category data not made widely available	data not accessible for public health decision-making.	The GTMP did this in each of its constituent projects.
5	(if the health ministry explicitly agreed), electronically transferred prevalence data to the International Trachoma Initiative, so that they could be used, where indicated, to justify applications for donated azithromycin for trachoma elimination purposes	manual transfer of data	increased effort, delays, errors.	GTMP data have leveraged a probable donation of 283 283 514 doses of azithromycin from Pfizer (including doses already donated, those approved for donation, and doses projected for future donation based on standard numbers of treatment rounds).

SUPPLEMENTAL REFERENCES

43. Mpyet C, Ogoshi C, Goyol M, 2008. Prevalence of trachoma in Yobe State, north-eastern Nigeria. *Ophthalmic Epidemiol 15*: 303-7.
44. Courtright P, Sheppard J, Schachter J, Said ME, Dawson CR, 1989. Trachoma and blindness in the Nile Delta: current patterns and projections for the future in the rural Egyptian population. *Br J Ophthalmol. 73*: 536-40.
45. Ndayishimiye O et al., 2011. Population-based survey of active trachoma in 11 districts of Burundi. *Ophthalmic Epidemiol 18*: 146-9.
46. Ngondi J, Onsarigo A, Adamu L, Matende I, Baba S, Reacher M, Emerson P, Zingeser J, 2005. The epidemiology of trachoma in Eastern Equatoria and Upper Nile States, southern Sudan. *Bull World Health Organ. 83*: 904-12.
47. Regassa K, Teshome T, 2004. Trachoma among adults in Damot Gale District, South Ethiopia. *Ophthalmic Epidemiol 11*: 9-16.
48. Solomon AW, 2003. Rational use of azithromycin in the control of trachoma: using quantitative PCR to assess distribution of infection and impact of treatment. Department of Infectious and Tropical Diseases. London: University of London, 259.
49. Hiep NX et al., 2017. Trachoma in Viet Nam: results of 11 surveillance surveys conducted with the Global Trachoma Mapping Project. *Ophthalmic Epidemiol* (In press).
50. Meng N, Seiha D, Thorn P, Willis R, Flueckiger RM, Dejene M, Lewallen S, Courtright P, Solomon AW, Global Trachoma Mapping Project, 2016. Assessment of Trachoma in Cambodia: Trachoma Is Not a Public Health Problem. *Ophthalmic Epidemiol*: 1-5.
51. World Health Organization Strategic and Technical Advisory Group on Neglected Tropical Diseases, 2018. *Design and validation of a trachomatous trichiasis-only survey (WHO/HTM/NTD/PCT/2017.08)*. Geneva: World Health Organization.
52. World Medical Association, 2013. World Medical Association Declaration of Helsinki: ethical principles for medical research involving human subjects. *JAMA 310*: 2191-4.
53. Trotignon G et al., 2017. The cost of mapping trachoma: Data from the Global Trachoma Mapping Project. *PLoS Negl Trop Dis 11*: e0006023.
54. Centers for Disease Control and Prevention, 2011. *Prevalence assessment of trachoma, Togo*. Altanta: Centers for Disease Control and Prevention.
55. Noa Noatina B, Kagmeni G, Mengouo MN, Moungui HC, Tarini A, Zhang Y, Bella AL, 2013. Prevalence of trachoma in the Far North region of Cameroon: results of a survey in 27 Health Districts. *PLoS Negl Trop Dis 7*: e2240.
56. Noa Noatina B, Kagmeni G, Souleymanou Y, Moungui HC, Tarini Hien A, Akame J, Zhang Y, Bella AL, 2014. Prevalence of trachoma in the north region of Cameroon: results of a survey in 15 health districts. *PLoS Negl Trop Dis 8*: e2932.
57. Yayemain D, King JD, Debrah O, Emerson PM, Aboe A, Ahorsu F, Wanye S, Ansah MO, Gyapong JO, Hagan M, 2009. Achieving trachoma control in Ghana after implementing the SAFE strategy. *Trans R Soc Trop Med Hyg 103*: 993-1000.
58. Dorkenoo AM, Bronzan RN, Ayena KD, Anthony G, Agbo YM, Sognikin KS, Dogbe KS, Amza A, Sodahlon Y, Mathieu E, 2012. Nationwide integrated mapping of three neglected tropical diseases in Togo: countrywide implementation of a novel approach. *Trop Med Int Health 17*: 896-903.
59. Southisombath K, Sisalermsak S, Chansan P, Akkhavong K, Phommala S, Lewallen S, Courtright P, Solomon AW, Global Trachoma Mapping Project, 2016. National Trachoma Assessment in the Lao People's Democratic Republic in 2013-2014. *Ophthalmic Epidemiol 23 (Suppl 1):* 8-14.
60. Kalua K, Chirwa T, Kalilani L, Abbenyi S, Mukaka M, Bailey R, 2010. Prevalence and risk factors for trachoma in central and southern Malawi. *PLoS One 5*: e9067.
61. Jip NF, King JD, Diallo MO, Miri ES, Hamza AT, Ngondi J, Emerson PM, 2008. Blinding trachoma in Katsina state, Nigeria: population-based prevalence survey in ten local government areas. *Ophthalmic Epidemiol 15*: 294-302.
62. Hassan A et al., 2011. The prevalence of blinding trachoma in northern states of Sudan. *PLoS Negl Trop Dis 5*: e1027.

63. Berhane Y et al., 2007. Prevalence of trachoma in Ethiopia. *Ethiop J Health Dev 21*: 211-5.
64. Kur LW et al., 2009. Trachoma in Western Equatoria State, Southern Sudan: implications for national control. *PLoS Negl Trop Dis 3*: e492.
65. Dolin PJ, Faal H, Johnson GJ, Ajewole J, Mohamed AA, Lee PS, 1998. Trachoma in The Gambia. *Br J Ophthalmol. 82*: 930-3.
66. Adera TH et al., 2016. Prevalence of and Risk Factors for Trachoma in Southern Nations, Nationalities, and Peoples' Region, Ethiopia: Results of 40 Population-Based Prevalence Surveys Carried Out with the Global Trachoma Mapping Project. *Ophthalmic Epidemiol 23*: 84-93.
67. Garn JV et al., 2018. Sanitation and water supply coverage thresholds associated with active trachoma: modeling cross-sectional data from 13 countries. *PLoS Negl Trop Dis 12*: e0006110.
68. Boisson S, Engels D, Gordon BA, Medlicott KO, Neira MP, Montresor A, Solomon AW, Velleman Y, 2016. Water, sanitation and hygiene for accelerating and sustaining progress on neglected tropical diseases: a new Global Strategy 2015-20. *Int Health. 8 Suppl 1*: i19-i21.
69. Mpyet C et al., 2016. Trachoma Mapping in Gombe State, Nigeria: Results of 11 Local Government Area Surveys. *Ophthalmic Epidemiol 23*: 406-411.
70. Kalua K, Chisambi A, Chinyanya D, Kamwendo Z, Masika M, Willis R, Flueckiger RM, Pavluck AL, Solomon AW, Global Trachoma Mapping Project, 2016. Completion of Baseline Trachoma Mapping in Malawi: Results of Eight Population-Based Prevalence Surveys Conducted with the Global Trachoma Mapping Project. *Ophthalmic Epidemiol 23 (Suppl 1)*: 32-38.
71. Mpyet C et al., 2016. Prevalence of Trachoma in Katsina State, Nigeria: Results of 34 District-Level Surveys. *Ophthalmic Epidemiol 23*: 55-62.
72. Omar FJ et al., 2016. Baseline Trachoma Surveys in Kaskazini A and Micheweni Districts of Zanzibar: Results of Two Population-Based Prevalence Surveys Conducted with the Global Trachoma Mapping Project. *Ophthalmic Epidemiol 23 (Suppl 1)*: 412-417.
73. Mpyet C et al., 2016. Prevalence of Trachoma in Bauchi State, Nigeria: Results of 20 Local Government Area-Level Surveys. *Ophthalmic Epidemiol 23*: 39-45.
74. Elshafie BE et al., 2016. The Epidemiology of Trachoma in Darfur States and Khartoum State, Sudan: Results of 32 Population-Based Prevalence Surveys. *Ophthalmic Epidemiol 23*: 381-391.
75. Adamu Y et al., 2016. Prevalence of Trachoma in Benishangul Gumuz Region, Ethiopia: Results of Seven Population-Based Surveys from the Global Trachoma Mapping Project. *Ophthalmic Epidemiol 23*: 70-76.
76. Sherief ST et al., 2016. The Prevalence of Trachoma in Tigray Region, Northern Ethiopia: Results of 11 Population-Based Prevalence Surveys Completed as Part of the Global Trachoma Mapping Project. *Ophthalmic Epidemiol 23*: 94-99.
77. Muhammad N et al., 2016. Mapping Trachoma in Kaduna State, Nigeria: Results of 23 Local Government Area-Level, Population-Based Prevalence Surveys. *Ophthalmic Epidemiol 23*: 46-54.
78. Adamu MD et al., 2016. Prevalence of Trachoma in Niger State, North Central Nigeria: Results of 25 Population-Based Prevalence Surveys Carried Out with the Global Trachoma Mapping Project. *Ophthalmic Epidemiol 23*: 63-69.
79. Abashawl A et al., 2016. Prevalence of Trachoma in Gambella Region, Ethiopia: Results of Three Population-Based Prevalence Surveys Conducted with the Global Trachoma Mapping Project. *Ophthalmic Epidemiol 23*: 77-83.
80. Bio AA et al., 2017. Prevalence of trachoma in northern Benin: results from 11 population-based prevalence surveys covering 26 districts. *Ophthalmic Epidemiol 24*: 265-73.
81. Haddad D, Nwobi B, Schmidt E, Courtright P, 2016. Elimination of Trachoma-Knowing Where to Intervene. *Ophthalmic Epidemiol 23*: 345-346.
82. Smith J, Mann R, Haddad D, Polack S, Kurylo E, Brooker S, 2016. *Global Atlas of Trachoma: an open-access resource on the geographical distribution of trachoma (www.trachomaatlas.org)*. Atlanta: International Trachoma Initiative.
83. Smith JL, Haddad D, Polack S, Harding-Esch EM, Hooper PJ, Mabey DC, Solomon AW, Brooker S, 2011. Mapping the global distribution of trachoma: why an updated atlas is needed. *PLoS Negl Trop Dis 5*: e973.
